# Variation in Meiofaunal Abundance and Composition Across an Estuarine Bay: Do They Comprise a Coherent and Distinct Entity Displaying Contrasting Patterns to the Macrofauna?

**DOI:** 10.1002/ece3.74076

**Published:** 2026-07-25

**Authors:** R. S. K. Barnes

**Affiliations:** ^1^ Institute for Coastal and Marine Research Nelson Mandela University Gqeberha Republic of South Africa; ^2^ Department of Zoology and Conservation Research Institute University of Cambridge Cambridge UK

**Keywords:** biodiversity, estuary, macrofauna, meiofauna, seagrass, South Africa

## Abstract

Patterns of meiofaunal distribution, abundance and composition were investigated across the disparate regions of the warm‐temperate Knysna estuarine bay, South Africa's premier site for estuarine biodiversity and conservation. This locality was represented by five seagrass and five adjacent bare‐sediment sites for which earlier data on the equivalent macrofaunal patterns were available. Thus it was possible to compare meiofaunal and macrofaunal responses to the same suite of contrasting habitats. Nematodes, copepods and ostracods, totalling 95.7% of numbers, dominated the Knysna epibenthic meiofauna which showed low (but not necessarily locally atypical) abundance: per core sample values of 19–886 (mean 211) ind. 10 cm^−2^. Meiofaunal abundance was subequal or greater in seagrass beds than in bare sediment, and peaked in the clean delta sands of the mouth. Each major component benthic taxon, whether macrofaunal or meiofaunal, responded individually to the estuarine gradients concerned and no common within‐group or contrasting between‐group macrofaunal or meiofaunal responses were apparent. Thus the macrofauna and meiofauna did not appear to behave as distinct and discrete ecological entities, as has been claimed. The macrofaunal ‘opportunistic polychaete to amphipod’ and meiofaunal ‘nematode to copepod’ ratios, used as indices of ecological quality, yielded differing (but in both cases unrealistic) results.

## Graphical abstract

Is division of marine animals into macrofauna and meiofauna merely an arbitrary convenience, or, as has been claimed, does their size difference render them distinct and independent entities evolved under different ecological constraints? Those in the Knysna estuarine bay did not behave as distinct and discrete ecological entities, their individual component taxa displaying disparate patterns independent of their allocation into the two size classes.

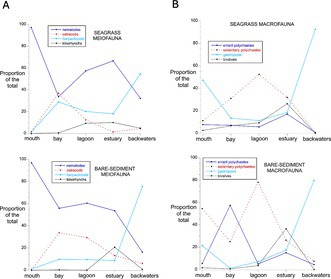

## 1 Introduction

An animal's body size is one of its most fundamental properties, affecting all aspects of its biology (LaBarbera 1989) and how we perceive it (Martínez et al. 2025). It has, for instance, for long been traditional to distinguish two groupings of aquatic animals living on/in the bottom sediments purely on the basis of their size: the macrofauna (those retained by a 0.5–1.0 mm mesh) and the meiofauna (microscopic forms < 500 μm but retained by a 32–63 μm mesh) (Giere 2014). To what extent is this division merely an arbitrary convenience, reflecting whether or not they can be easily discriminated by human sight and on the necessarily different techniques required for their investigation? Yamanaka et al. (2012), measuring body size of the benthos of a series of sandy beaches in the UK, found a bimodal distribution with a consistent trough at 0.5–1.0 mm separating the two peaks, suggesting a real difference between the two categories and indicating that they might form natural rather than artificial groupings. Others (e.g., Armenteros et al. 2007; Adão 2021; Chertoprud and Novichkova 2023; Kim et al. 2023) have also suggested that marine macrofauna and meiofauna are influenced by and respond to different environmental factors and thus are more than pragmatically distinct, the meiofauna comprising ‘an independent structural unit … with its own spatial and temporal characteristics’ (Chertoprud and Novichkova 2023, 1), and representing ‘a separate group of animals … with a coherent life history and feeding characteristics, which sets them apart as a separate evolutionary unit’ (Adão 2021, 695). Certainly, in relatively coarse substrata there is a clear distinction between animals capable of living in interstitial spaces (Swedmark 1964) and those displacing the sedimented particles when moving; in finer grades, however, this is less so. But other size‐based discontinuities also occur within the macrofaunal category (Jacob et al. 2011; Yamanaka et al. 2012) without thereby being considered to distinguish separate faunal or ecological components. Is the distinction between macrofauna and meiofauna somehow more valid?

There is indeed little overlap in the major taxa dominating the two size classes (at least when adult): they are naturally distinguished at least taxonomically. The most numerous meiofaunal animals include nematodes, harpacticoid copepods, ostracods, halacarid mites, and various groups of ‘turbellarian’ and ‘archiannelid’ worms (Giere 2014; Schmidt‐Rhaesa 2020). Effectively, only parasitic members of these taxa (if any) are macroscopic. Several whole phyla, such as the Gastrotricha, Gnathostomulida, Kinorhyncha, Loricifera, and Tardigrada, are only meiofaunal. In marked contrast, few of the taxa dominating the macrofauna are also successfully meiofaunal. Granted the advantages of vermiform shape for interstitial existence, it is not surprising that annelids do span the divide, but they too are usually a minor component of the meiofauna, comprising only some 2% of the total (Worsaae et al. 2021).

If the two size categories do respond differently to environmental variables, they might be predicted to diverge in their responses to those characterising a shared system, yielding characteristic macrofaunal and meiofaunal patterns of distribution and abundance. Whether this is so has yet to be tested (Magni et al. 2022) but localities such as the Knysna estuarine bay in warm‐temperate South Africa (34°03′S,23°03′ E) could form useful sites for such testing. This system supports the richest macroscopic estuarine biodiversity in South Africa (Turpie et al. 2002; Turpie and Clark 2007), and is arguably one of the two most studied and hence best known coastal inlets in the country, with a well documented macrofauna that has been surveyed and re‐surveyed for the last 75 years (Whitfield et al. 2023). Notwithstanding its microtidal state and very narrow entrance channel (see Warwick et al. 2021), it comprises a structurally diverse series of soft‐sediment benthic habitats, ranging from exposed to sheltered, from vegetated to bare, from extensive tidal flats to steep‐sided channels, from surface cohesive mud to mobile tidal‐delta sand, and from those flooring the main axial channel to the beds of small peripheral, saltmarsh‐enclosed, backwater creeks. Accordingly, its macrofaunal assemblages display much spatial variation in their nature and abundance, not only regionally (see below) but, within each region, in relation to the presence or absence of beds of seagrass (Barnes 2021, 2022). Since meiofaunal composition and abundance are also known to vary with habitat type in estuaries and lagoons (Dye 2006; Sharma et al. 2022; Magni et al. 2022; Kim et al. 2023), the variety of Knysna habitats would therefore appear likely also to provide its meiofauna with the opportunity to express divergent regional abundance and composition, and, should the two groups really be distinct, to do so in a different manner to that/those shown by the macrofauna.

Nevertheless, despite its many years of scientific attention, study has been devoted only to some parts of the Knysna system and to some elements of its biota; as is often the case, smaller organisms including the meiofauna, receiving almost none. The present work therefore had two aims. (1) To establish the variation in meiofauna across contrasting sections of the Knysna system, concentrating on those assemblages present at the low tide level in both seagrass and bare sediment at the specific sites for which comparable recent data on the macrofauna are already available. (2) Thereby to compare the distribution, abundance and relative composition patterns displayed by the Knysna macrofauna and meiofauna, both for these two size‐based categories as a whole and for their major individual component taxa. In that regard, the following prediction was examined: that the distribution patterns of the meiofauna and macrofauna across the two different habitats (seagrass and bare sediment) and five notional system compartments of the Knysna system displayed consistent within‐group similarity but between‐group differences. The specific hypotheses tested were: that the patterns of abundance of the various groups of meiofauna were concordant, and likewise those of the macrofauna, but that the patterns of those two groupings differed. The data collected also provided an opportunity to compare assessment of the environmental status or ‘health’ of the different Knysna regions using macrofaunal and meiofaunal data, that is, the ‘opportunistic‐polychaete to sensitive‐amphipod’ ratio of Dauvin et al. (2016) (see Barnes and Seath 2025) and the ‘nematode to copepod’ ratio of Raffaelli and Mason (1981) (see Semprucci et al. 2016).

## 2 Methods

### 2.1 Study Sites

**FIGURE 1:**
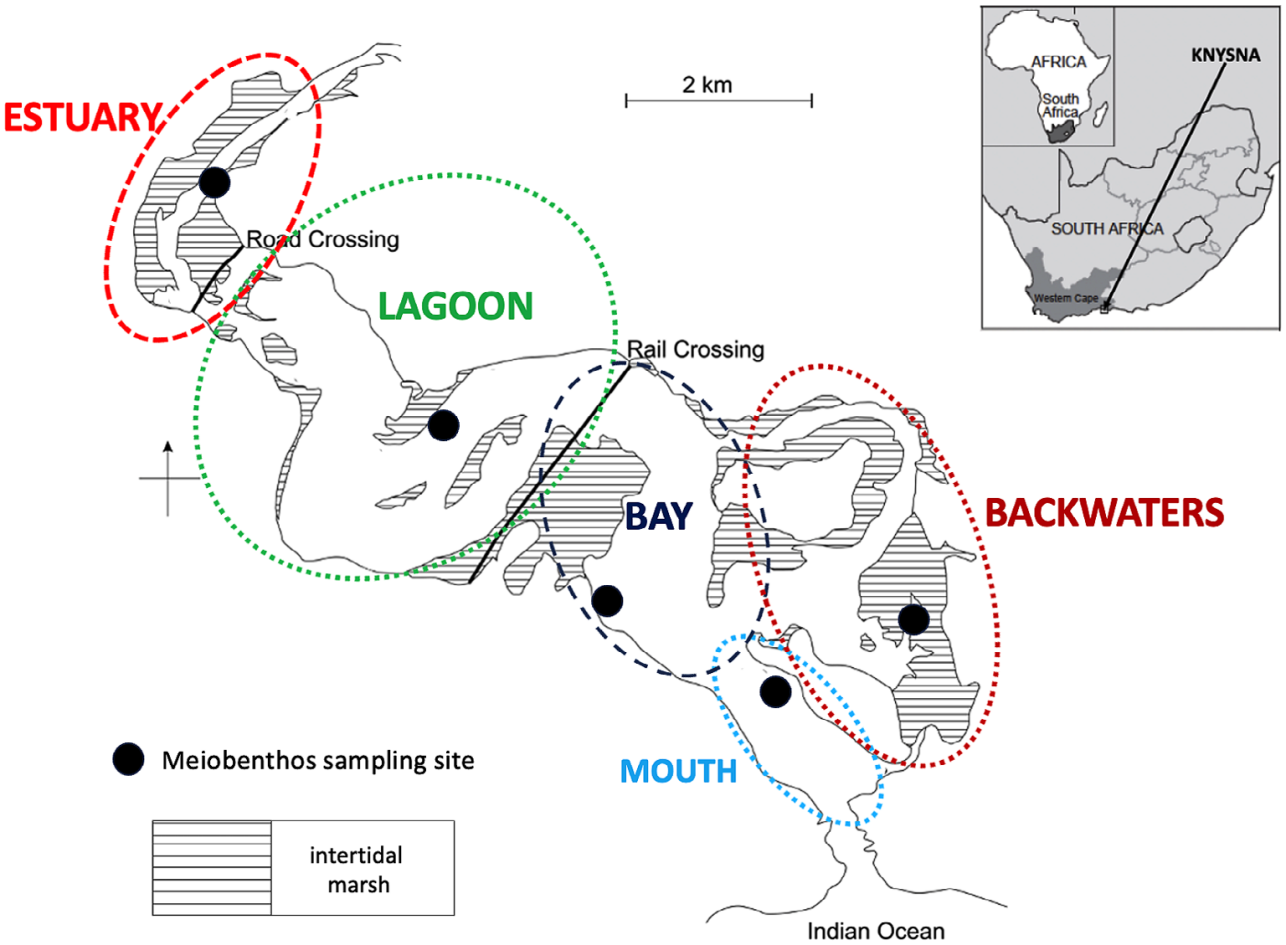
The Knysna estuarine bay and its five notional compartments (after Barnes and Seath 2025), with the meiofaunal (and earlier macrofaunal) sampling site in each compartment indicated.

The Knysna estuarine bay is a permanently‐open, seawater‐dominated, oligotrophic, warm‐temperate, drowned river valley located within the Garden Route National Park. It is mostly floored by sand blown in or swept in by the tide from the Tertiary‐Pleistocene fossil dune system enclosing it, but has admixed finer surface particles in its backwaters, river‐discharge zones and beds of macrophytes (Reddering and Esterhuysen 1987; Cooper and Green 2023). Most of its shallow‐water soft sediments are structured by the gebiidean mudprawn *Upogebia*. In respect of the other macrofauna, it can be considered to comprise five notional ‘compartments’ (Figure 1), four in a linear chain along its longitudinal axis from the extensive flood‐tidal sand delta at its mouth to the narrow muddy estuary of the Knysna river at its head, plus a fringing system of higher‐level, saltmarsh‐enclosed, backwater channels, creeks and pools sheltered between two large bay islands and the mainland. Although three of these five compartments change precise location dependent on tidal state and freshwater input, all display different hydrographical (Largier et al. 2000) and sedimentary (Reddering and Esterhuysen 1987) characteristics, and they support significantly different macrofaunal assemblages albeit with several shared species (Barnes 2021).

### 2.2 Sampling

To represent each of these five notional system compartments (Figure 1), twelve randomly‐located replicate epibenthic meiofaunal samples were taken from within (a) a seagrass bed (of Cape eelgrass, *Nanozostera capensis* sensu Sullivan and Short 2023) and from (b) a nearby area of bare sediment, the precise collection sites chosen all being ones at which the seagrass and bare‐sediment macrofauna had recently been determined (see below). All samples were taken whilst the surface of the substratum was covered by at least 10 cm of water, and all were located at least 2 m away from habitat interfaces to avoid any possible edge effects (Nakaoka 2005; Barnes and Hamylton 2016).

Coull (1999) considered that 1000 ind. 10 cm^−2^ could be taken as the expected abundance of estuarine meiofauna worldwide, although known lagoonal and estuarine meiofaunal densities range from > 8000 ind. 10 cm^−2^ (Castel 1992; Sharma et al. 2022) down to < 60 (Dye 1979; Nozais et al. 2005; Pillay and Perissinotto 2009). It is relevant to note that the three studies cited above as recording the lowest densities all refer to South African estuaries (including ciliates in the numbers in one case). On the basis of the Coull (1999) expectation, a core of 3.75 cm^2^ area would yield a total of some 375 individual animals, and that size of unit sample was adopted here. A 2 cm depth was selected so as to sample the epibenthos (similarly to Pillay and Perissinotto 2009), that is, the surface zone to which estuarine meiofauna are largely restricted (Sharma et al. 2022, and references cited therein). Core samples were taken during low tide in February and March for comparability with the late summer/early autumn season of the earlier macrofaunal sampling.

Contained meiofauna were extracted using the methodology of Cross and Curran (2000), and Armenteros et al. (2008). Each 7.5 cm^3^ core sample was first sieved through 500 μm mesh to remove macrofauna and was then placed in an Erlenmeyer flask to which *c*. 0.25 L freshwater was added, vigorously swirled, and the supernatant decanted after four seconds through 63 μm mesh. This filling/shaking/decanting sequence was repeated five times; after which the material retained in the 63 μm sieve was washed with a little local seawater into a petri dish with a 6 × 6 cell graticule and was viewed under a binocular microscope at *c*. ×20 magnification, the meiofauna observed being identified and counted. Meiofauna were thus examined alive, not least to facilitate inclusion of cuticle‐less non‐ecdysozoan taxa (Curini‐Galletti et al. 2020) that are often destroyed by treatment techniques and hence underestimated (Balsamo et al. 2020). When abundant organic debris including *Upogebia* faecal pellets was also present, a single sample was split between a number of such petri‐dishes. After enumeration the meiofauna were returned to their habitat.

Species identity of most South African coastal meiofauna being uncertain or unknown, animals were allocated to the following higher taxa or informal groups represented in the samples: ‘flatworms’, nematodes, kinorhynchs, gastrotrichs, ostracods, harpacticoids, and mites. Because of the specific nature of the comparison underlying the study, juvenile macrofauna were not included in counts of meiofauna (all oligochaetes were assessed as juvenile macrofauna, many relatively very large individuals passing through the 500 μm sieve). For comparison with the meiofauna, the earlier macrofaunal data of Barnes and Barnes (2014), Barnes and Claassens (2020) and Barnes (2022, 2025) were therefore also allocated to a series of equivalent higher taxa: polyclads + nemertines, errant polychaetes, sedentary polychaetes (including oligochaetes), the large ostracod? *Cylindroleberis*, peracaridans, decapods, gastropods, bivalves, and echinoderms (see Appendix A). This coarse scale of taxonomic analysis is widely considered sufficient for ecological studies of the present type (Pillay and Perissinotto 2009; Semprucci et al. 2019; Sharma et al. 2022). *N.B*. Some usages of the term meiofauna include organisms other than animals, for example, some, but by no means all, members of the kingdom Chromista (most often the phyla Foraminifera and Ciliophora). That usage was not followed here. All animal taxa are as listed in the World Register of Marine Species (www.marinespecies.org) accessed March 2026.

### 2.3 Analyses

Numbers per unit area of each component meiofaunal taxon were subjected to similarity analysis, and assemblage metrics were derived and compared via *PAST* 5.2 multivariate software (Hammer et al. 2001) or Microsoft Excel for Mac 16.106 with the StatPlus:mac Pro 8.0.4 add‐on, all metrics being based on numerical abundance. Meiofaunal composition data showed unequal multivariate dispersion across notional system compartments and habitats (PERMDISP *F* > 9; *p* < 0.006) and, being very robust to unequal dispersion if sample sizes are balanced and if the data are in Euclidean form (Anderson and Walsh 2013), PerMANOVA was used in such multivariate comparisons with 9999 iterations. Multivariate comparisons and hierarchical classification were both conducted after Hellinger transformation of the raw data because of the marked dominance of some taxa. Differences in local abundance were tested by Mann–Whitney *U* tests and in rank orders by Kendall's tau (*τ*) concordance.

## 3 Results

### 3.1 Distribution and Abundance of Meiofauna

The observed abundance of meiofauna across the ten Knysna sites sampled (the five notional compartments each with two habitat types) is shown in Table 1. This displays domination by nematodes (72.2% of individuals sampled), harpacticoids (15.1%), ostracods (7.4%) and kinorhynchs (3.7%). Nematodes were the most abundant group in seven out of the ten individual sites, but the various other dominant non‐nematode taxa were each most abundant in differing areas. Harpacticoids were most numerous in both the seagrass and bare sediment of the backwaters site. Ostracods were most numerous in those of the bay. Kinorhynchs were commonest in the seagrass and bare sediment of the estuary. There was no significant positive or negative concordance between the abundances of these three dominant taxa (*τ* = < 0.2, *p* > 0.4), so that there was no unitary ‘meiofaunal response’ to the Knysna gradients amongst its component taxa and nothing to suggest that the meiofauna behaved as a distinct unit. Although not significantly so, the abundance of nematodes was negatively related to those of both ostracods and harpacticoids (*τ* = <−0.45, *p* > 0.2). Flatworms, mites and gastrotrichs were uncommon (each < 1.5% of numbers), and none of the interstitial polychaete groups (sensu Worsaae et al. 2021) were observed although a psammodrilid is known to occur in the delta sands at the mouth (Barnes 2025). Mean meiofaunal abundance was only 211 ind. 10 cm^−2^, with a per‐site range of 42 ind. 10 cm^−2^ (in the bare sediment of the lagoon) to 537 ind. 10 cm^−2^ (in the seagrass at the mouth).

**TABLE 1:** Number of meiofauna 10 cm^−2^ at the ten disparate sites representing the Knysna estuarine bay (based on 12 core samples in each case).

Compartment	Habitat	Nem	Cop	Ost	Kin	Fla	Hal	Gas
Mouth	Seagrass	521	7	9	< 1	0	0	0
Mouth	Bare	473	5	1	0	8	< 1	< 1
Bay	Seagrass	46	39	50	< 1	0	0	0
Bay	Bare	41	7	25	0	< 1	0	0
Lagoon	Seagrass	128	43	27	20	< 1	< 1	0
Lagoon	Bare	25	4	12	< 1	< 1	0	0
Estuary	Seagrass	159	43	3	24	10	0	0
Estuary	Bare	70	11	17	27	1	4	0
Backwaters	Seagrass	40	67	6	6	4	0	0
Backwaters	Bare	20	93	8	1	2	0	0
Overall mean value		152	32	16	8	3	< 1	< 1

Abbreviations: Cop, harpacticoid copepods; Fla, ‘flatworms’; Gas, gastrotrichs; Hal, halacarid mites; Kin, kinorhynchs; Nem, nematodes; Ost, ostracods.

**FIGURE 2:**
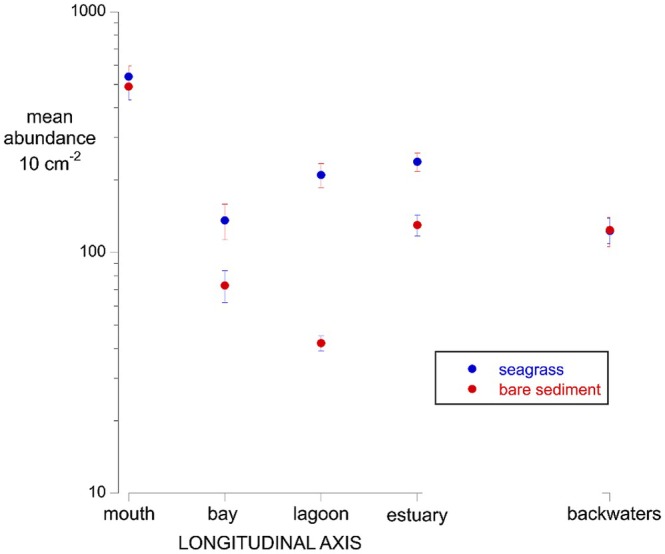
Variation in overall meiofaunal abundance (mean values per 10 cm^2^ ± standard error) along the longitudinal channel of the Knysna estuarine bay (mouth, bay, lagoon, and estuary compartments) and in its backwaters, both in seagrass beds and in adjacent areas of bare sediment.

Two‐way PerMANOVA of assemblage composition showed a significant interaction between system compartment and habitat type, and hence one‐way simple‐effect analyses were conducted instead. The five compartments supported significantly different meiofaunal assemblages both in their seagrass systems and in their adjacent areas of bare sediment (PerMANOVA *F* > 18, *p* < 0.003) in all comparisons, with the exception of the bare sediment meiofaunal assemblages of the bay and lagoonal compartments (*F* = 0.8, *p* = 0.46). Seagrass and bare sediment habitats likewise supported significantly different assemblages in each compartment (PerMANOVA *F >* 5.05, *p* < 0.002 in all cases), although overall the assemblages of the two habitat types were not dissimilar (PerMANOVA *F* > 2.1, *p* = 0.1). As a habitat, all but one sampled seagrass bed supported greater abundance than adjacent bare sediment in a ratio of 1.1–5.0 (Table 1), although only marginally and not significantly so at the mouth (overall Mann–Whitney *U =* 1050, *p* < 0.0001). The two habitat types at the backwaters site supported equal numbers. The same differential also applied to most individual component taxa, although flatworms at the mouth, and both ostracods and mites in the estuary bucked the trend. The mouth compartment supported the most meiofauna by a large margin and the bay one the least (Figure 2). Meiofaunal abundance thus did not show a simple relationship with the upstream gradient along the main channel, but numbers per core sample nevertheless did decrease upstream (*τ* = 0.26, *p* = 0.0006).

**FIGURE 3:**
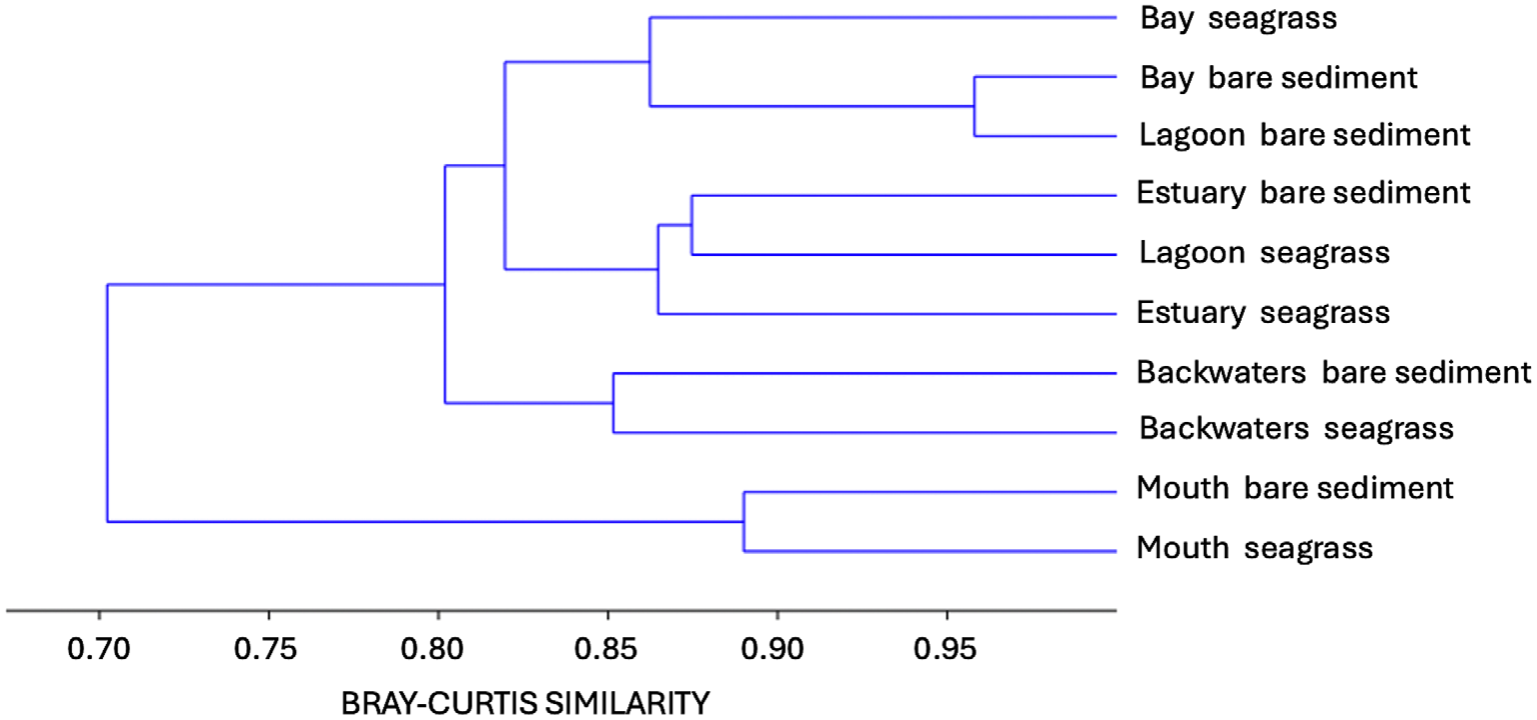
Pattern of Bray–Curtis similarity in quantitative meiofaunal assemblage composition between the various notional compartments and their two habitat types in the Knysna estuarine bay using Hellinger‐transformed abundance data.

Similarity between the various sites in terms of their meiofaunal assemblages is displayed in Figure 3: the clean delta sand of the mouth compartment with its (relatively) abundant nematodes and scarce other fauna stood apart from the others, as to a slightly lesser extent did the backwaters which display the converse states of low nematode numbers but relatively abundant other groups. The other sites segregate by a mixture of upstream‐gradient and habitat‐type characteristics, neither variable predominating.

### 3.2 Corresponding Distribution and Abundance of Macrofauna

**FIGURE 4:**
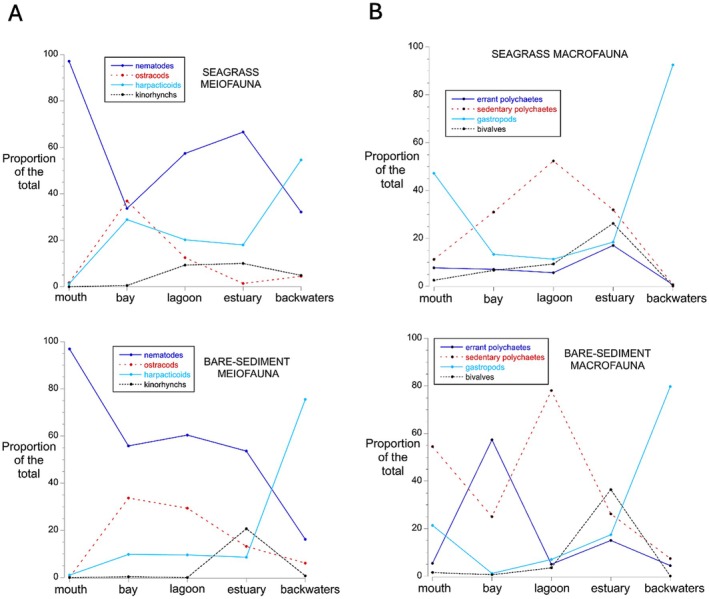
Relative frequencies of the four most abundant meiofaunal (A) and macrofaunal (B) taxa in the seagrass and adjacent bare‐sediment habitats across the different notional compartments of the Knysna estuarine bay.

The relative overall abundances of Knysna's benthic macrofaunal taxa at the same ten sites as investigated here in respect of their meiofauna, and those of the meiofauna themselves, are set out in Table 2 and in the Appendix A. Table 2 clearly shows that, like the meiofauna, there is a very wide range of individual values of local abundance for the macrofauna, with a maximum some 23 times the minimum, although their ranked relative abundances take the same general form in each compartment (see Barnes 2021; Figure 4). Together, on average, polychaetes, gastropods, and bivalves comprised 84% of Knysna's macrofauna across both habitats and the five compartments, and hence it is their patterns that largely determine those of the macrofauna as a whole.

**TABLE 2:** Comparison of the relative abundance and (parenthetically) rank order of the macrofaunal and meiofaunal assemblages at the ten disparate sites representing the Knysna estuarine bay (macrofaunal data from the databases listed in the Appendix).

Compartment	Habitat	Meiofauna	Macrofauna
Mouth	Seagrass	12.79 (10)	4.55 (8)
Mouth	Bare	11.62 (9)	1.82 (2)
Bay	Seagrass	3.24 (6)	3.61 (6)
Bay	Bare	1.76 (2)	3.66 (7)
Lagoon	Seagrass	3.21 (5)	3.21 (5)
Lagoon	Bare	1	2.72 (4)
Estuary	Seagrass	5.57 (8)	2.36 (3)
Estuary	Bare	3.10 (5)	1
Backwaters	Seagrass	2.93 (2=)	12.94 (9)
Backwaters	Bare	2.93 (2=)	23.37 (10)

Although the species concerned were not the same, gastropods as a taxon dominated the seagrass both in the backwaters and mouth regions, whilst sedentary polychaetes dominated those in the lagoon. It was likewise gastropods that dominated the bare sediment of the backwaters, and sedentary polychaetes that dominated those in the mouth and lagoon. Bivalves were dominant in the estuary and errant polychaetes were so in the bay (Figure 4). Amongst the more important component taxa, only the abundance of gastropod molluscs was significantly concordant with those of any other group, with both peracaridan crustaceans and decapod crustaceans (*τ* = > 0.55, *p <* 0.03). Therefore there is little to suggest that the macrofauna comprised a distinct ecological unit either. Indeed, along the axial channel significant concordance of component abundances cut across the meiofaunal/macrofaunal division with the only other significant pairing being between numbers of gastropods and nematodes (*τ* = 0.98, *p* < 0.04). Different groups of both the meiofauna and macrofauna were numerically dominant in different regions (Table 3) in a pattern generally devoid of overall similarity across regions. Only the adjacent seagrass sites of the bay and lagoon compartments and the two habitat types in the estuary showed significantly similar faunal composition (*τ* = 0.60 and 0.65, *p* < 0.03 and < 0.007, respectively). Comparison of meiofaunal and macrofaunal group patterns indicates that a common feature was that both showed peak abundance in the mouth region, but they differed in that macrofauna were more abundant in backwaters than along the main estuarine channel whereas meiofauna although relatively common did not peak there. Overall, the abundance of both groups decreased along the upstream axial gradient (*τ* = 0.36, *p* > 0.2).

**TABLE 3:** Rank orders of relative abundance of the major component macrofaunal and meiofaunal taxa at each of the ten representative sites representing the Knysna estuarine bay (macrofaunal data from the databases listed in the Appendix).

	Mouth		Bay		Lagoon		Estuary		Backwaters	
	Grass	Bare	Grass	Bare	Grass	Bare	Grass	Bare	Grass	Bare
*Meiofauna*										
Nematodes	1	2	6	7	4	9	3	5	8	10
Copepods	6	10	1	3	2	5	9	4	8	7
Ostracods	8	9	5	7	4	10	3	6	2	1
Kinorhynchs	—	7=	6	7=	3	—	2	1	4	5
*Macrofauna*										
?*Cylindroleberis*	2	4	1	6	3	5	—	—	—	—
Peracaridan crustaceans	3	2	5	7	6	8	9	10	4	1
Decapod crustaceans		6	4	9	2	7	8	10	3	5
Errant polychaetes	4	3	8	2	6	7	5	9	10	1
Sedentary annelids	7	1	5	8	3	2	6	9	10	4
Gastropod molluscs	3	4	6	10	5	8	7	9	2	1
Bivalve molluscs	7	6	4	10	2	5	1	3	9	8

### 3.3 Comparison of Macrofaunal and Meiofaunal Indices of Benthic Ecological Quality

Ratios of the abundance of (a) macrofaunal opportunistic polychaetes versus amphipods and of (b) meiofaunal nematodes versus copepods across the seagrass and bare‐sediment habitats in the five notional compartments of the Knysna estuarine bay are set out in Table 4. There was no concordance between the two rank orders (*τ* = 0.24; *p =* 0.4). The inferences associated with use of the meiofaunal nematode: harpacticoid ratio (Hennig et al. 1983; Rubal et al. 2009) would suggest most organic enrichment at the mouth, and least at the backwaters. It was thus particularly notable that using that ratio as a guide, the most pristine part of the system, the region swept by large tidal fluxes twice daily and floored by clean mobile delta sand, would be ranked the most organically enriched. The organically‐enriched, eutrophic backwaters compartment (Largier and Human 2023) would be assessed as the least (Table 1)! The macrofaunal ratio presents comparable problems (Barnes and Seath 2025), so neither appear at all useful in the Knysna system.

**TABLE 4:** Comparison of the values and (parenthetically) rank order of the macrofaunal ‘opportunistic‐polychaete to sensitive‐amphipod’ (*Pol/Amp*) ratio and the meiofaunal ‘nematode to copepod’ (*Nem/Cop*) ratio at the ten disparate sites representing the Knysna estuarine bay.

Compartment	Habitat	Pol/Amp ratio	Nem/Cop ratio
Mouth	Seagrass	0.76 (2)	74.4 (9)
Mouth	Bare	2.27 (5)	94.6 (10)
Bay	Seagrass	1.60 (3)	0.91 (3)
Bay	Bare	2.21 (4)	5.69 (6)
Lagoon	Seagrass	15.33 (7)	2.99 (4)
Lagoon	Bare	48.52 (10)	6.25 (7)
Estuary	Seagrass	20.92 (8)	3.70 (5)
Estuary	Bare	29.0 (9)	6.26 (8)
Backwaters	Seagrass	5.40 (6)	0.60 (2)
Backwaters	Bare	0.54 (1)	0.21 (1)

## 4 Discussion

### 4.1 Meiofaunal Patterns

Nematodes are the dominant meiofauna in most South African estuaries in which that size range has been examined, averaging some 75%–80% of total numbers; for example, in the Swartzkop (Dye and Furstenberg 1978), Gamtoos (Gyedu‐Ababio 2011), Mngazana (A. H. Dye 1983), and St Lucia (Bownes and Perissinotto 2012). Indeed they dominate estuarine meiofauna throughout the world, attaining 22,680 ind. 10 cm^−2^ in the Lynher Estuary in the UK (Warwick and Price 1979). Harpacticoids usually occupy second place. Thus, in its domination by nematodes and harpacticoids, the Knysna meiofauna is that of a typical estuary, and, again as elsewhere (Sørensen and Pardos 2008), its relatively abundant kinorhynchs are mainly associated with sediments containing admixed fine particles. At face value the rarity of gastrotrichs is perhaps more unusual, in that they are regarded as being characteristically third in importance after nematodes and harpacticoids, and even in some instances the most or second‐most numerous taxon in shallow marine sediments (Todaro and Hummon 2008). They do however seem to prefer relatively coarse sands, whilst those in Knysna are basically fine, and they have not been commonly observed in other South African estuaries either. Few other meiofaunal taxa are known to achieve local dominance, and, if they do, do so only in some samples or at some times. Mites, for example, were a dominant group in the temporarily open/closed Mdloti estuary, especially during the same summer months as those in which the Knysna survey was conducted. Then overall they accounted for more than a third of total numbers (Nozais et al. 2005). Flatworms can also be a sub‐dominant group, as in some Swartkops samples (Gyedu‐Ababio 2011). Each of these groups was present in Knysna but only in a few samples, but in them could be represented by considerable numbers. The relative importance of ostracods at several sites, dominating the bay seagrass for example, does seem a particular characteristic of Knysna. Besides their relative meiofaunal importance, the large (up to 3 mm)? *Cylindroleberis* is a dominant member of its macrofauna, and several new benthic species have been described from there (Benson and Maddocks 1964).

The abundance of its meiofauna, however, appears low. The overall mean Knysna density of 211 ind. 10 cm^−2^ (with a maximum per‐site value of 537 ind. 10 cm^−2^) is clearly well below the general expectations of Coull (1999) and Giere (2014), for example. Values at individual sites were relatively consistent, however, so although meiofauna are often patchy (Findlay 1981) this would not appear to have influenced this result. Neither is it likely that considering only the epibenthic forms is the cause, granted that studies of variation in meiofaunal abundance with depth in the sediment almost always suggest that > 50% of animals occur in the top 2 cm. There may, however, be two reasons for the apparently low values. First, oligotrophic systems are known to support fewer than usual meiofauna (Bianchelli et al. 2020) and Knysna is oligotrophic, especially so in the bay and lagoonal compartments (Switzer 2003; Largier and Human 2023). Secondly, and perhaps most importantly, Dye (1977), conducting two unpublished transects down the Knysna intertidal zone (Urban‐Malinga 2014), recorded maximum meiofaunal abundance above mean tide level. Densities near the low tide mark were 15–18 × less than near the top of the shore, at < 120 ind. 10 cm^−2^. McLachlan et al. (1981) also recorded general meiofaunal abundance on sandy shores in southern Africa as declining rapidly from mid to low water level. These somewhat counterintuitive findings for intertidal marine organisms presumably relate to the location of peak microphytobenthic productivity at mid‐shore levels (see, e.g., Underwood 2001). Nevertheless, although the Knysna densities may be low by world standards, they appear well within the typical range for South Africa, at least for the tidal height at which they were collected, and are comparable with some from the Mediterranean, Red Sea and elsewhere (e.g., Moreno et al. 2006; El‐Serehy et al. 2015). For the purpose of the intended comparison, the season and tidal height of the present study were set to correspond to those of the earlier macrofaunal sampling, itself chosen to be at a time and zone when/where macrofaunal densities were high (e.g., Kaletja and Hockey 1991) following the austral spring breeding season (Hodgson 2010). This may therefore have had the consequence of sampling at a tidal height of relatively low meiofaunal abundance. Even if that was so, however, there is no reason to believe that this will have resulted in aberrant overall patterns of meiofaunal distribution, assemblage composition or relative abundance from region to region within the estuarine bay.

### 4.2 Macrofaunal Versus Meiofaunal Patterns

Considering the distribution and abundance of the Knysna meiofauna as a whole, two features stand out. Overall, its abundance in seagrass was greater (1.5×) than in adjacent areas of bare sediment. This is often, although not universally, the case. It is, for example, more abundant in various French, Taiwanese, and Chinese seagrass localities (Castel et al. 1989; Liao et al. 2016; Liu et al. 2025), but not so in New South Wales, Australia (Fonseca et al. 2011). The same generality also applies to the macrofauna. Species of *Zostera* and *Nanozostera* classically support a greater macrofauna than does adjacent bare sediment (Kindeberg et al. 2022; Colvin and Snelgrove 2025; etc.). Not markedly so through most of the Knysna system, however, and such differential as there is depends on locality (Barnes 2022). In some areas, the opposite is even the case (Barnes and Barnes 2014; and see Xu et al. 2018).

The second noteworthy feature concerns its relative abundance along the upstream estuarine gradient. The typical meiofaunal pattern is for numbers to decrease upstream (Coull 1999; Alves et al. 2009), much as in the estuarine macrofauna (Whitfield et al. 2012). In Knysna, maximum meiofaunal abundance did indeed occur at the mouth, and although overall it did decrease along the longitudinal upstream gradient, it did not do so uniformly. It was least in the two oligotrophic central zones of the bay and lagoon, the lagoonal compartment in particular having long water‐residence times and being mixed more by tidal diffusion than by flushing (Largier et al. 2000). Similar atypical meiofaunal distribution patterns with minimal abundance in the middle reaches of transitional situations are, however, known elsewhere in the warm‐temperate zone (Yamamuro 2000). The mouth of the Knysna system being clean tidal‐delta sand means that the maximum meiofaunal abundance at the estuarine mouth overrode another distributional expectation—that the meiofauna should be least numerous in clean sand (Coull 1999).

The somewhat unusual detailed pattern of meiofaunal upstream abundance, however, did not overall differ significantly from the more regular decline usually demonstrated by the Knysna macrofauna (Barnes 2019, 2022). Abundance of the two faunas could thus be regarded as responding in a broadly similar manner to the same environmental upstream variables, as also found by Rubal et al. (2011) in a north‐west Spanish ria and by Magni et al. (2022) in the Sardinian Cabri Lagoon. In essence, abundance of estuarine animals responds to the gradients concerned in a standard fashion, irrespective of their individual sizes. Macrofaunal abundance in the backwaters, however, did contrast markedly with that of the meiofauna in being very large in relation to that along the longitudinal axis (Barnes and Barnes 2014). This is not always the case, however. When present, the effect is largely caused by extreme local abundance of the endemic epifaunal gastropods *Davishydrobia* and *Davisassiminea* that are dominant in the backwater zone at some times, as they were in 2014 (Barnes and Barnes 2014), but not at others, for example in 2018 (Barnes 2018). The same two truncatelloids also fluctuate in abundance in the estuarine system compartment, sometimes dominating the macrofauna there (Barnes and Ellwood 2012) and sometimes effectively being absent (Barnes 2022). This does appear to be characteristic of such microgastropods (Barnes 1991; Haubois et al. 2002). As this is the only survey of the Knysna meiofauna that has been conducted, information on local interannual temporal variability in animals of that size range for comparison is not available, although such is known to occur elsewhere (Vieira et al. 2025).

The macrofaunal data from Knysna presented above clearly indicate that individual taxa are abundant in different sections of the longitudinal gradient, although polychaetes maintained high density through all but the most brackish zones (Barnes and Seath 2025). There is no sense, however, that the macrofaunal taxa show some characteristic ‘macrofaunal pattern’ of relative abundance. Each taxon is distributed in a manner appropriate to the autecology of the type of animal concerned. Precise patterns of individual taxa are known to vary not only from region to region but from estuary to estuary dependent on substratum, vegetation, etc. (Barnes 1989; Laurino et al. 2021). Nematodes dominate the meiofauna across most regions of the Knysna longitudinal axis in a manner that no taxon amongst the macrofaunal groups does; numerically they are *the* dominant benthic taxon throughout the whole estuarine system, and to a large degree their pattern is therefore that of the whole meiofauna. The contribution of the other meiofaunal taxa, however, like those of the macrofaunal groups, does vary from region to region in Knysna and, granted that there is no positive correlation between the abundances of the different component groups, there is no characteristic pattern in relative distribution or abundance of the whole meiofauna either, except as dictated by the one overwhelming nematode taxon. In estuarine and lagoonal environments generally, the dominant benthic animals (the meiofaunal nematodes, harpacticoids, ostracods and kinorhynchs, and macrofaunal sedentary polychaetes, peracaridans and gastropods) are all members of groups known to be essentially deposit feeders. They are all dependent on the detrital organics, bacteria and microphytobenthos associated with the sediment on or in which they live (Giere 2014; Lopez et al. 2024). In that respect they are certainly not independent ecological units. It is therefore hardly surprising that taxonomic group of the Knysna fauna, each of which has its own characteristic method of acquiring and processing this resource, should be a more important differentiating factor in their local patterns of distribution and abundance than whether they are larger or smaller than 0.5 mm.

Macrofaunal and meiofaunal distributions across the estuarine bay did display some differences, their differential reaction to the backwater and estuarine compartments being the most marked. As discussed above, however, these seem entirely dependent on whether the highly variable macrobenthic trunctatelloid gastropods *Davishydrobia* and *Davisassiminea* were in boom or bust phase. In any event, the relevant question is: in their distribution and abundance, were any meiofaunal and macrofaunal differences attributable to them being larger or smaller than 0.5 mm? This survey uncovered no evidence that this was the case. Such is the variation in the distribution of individual taxa shown in Figure 4, for example, that it would appear highly unlikely that any two distribution patterns drawn from random subsets of taxa from the whole Knysna zoobenthos would display identical patterns, and macrofaunal and meiofaunal differences must be viewed in that light. Other possible subsets of taxa are likely to show differences of equivalent or even greater magnitude.

These data clearly have their limitations, not least because each notional region was represented by only single paired seagrass and bare‐sediment sites. Nevertheless, in the absence of evident shared within‐group and/or contrasting between‐group distributional or abundance characteristics, patterns in the Knysna system do not lend support to notions that the various individual taxa that can, on the basis of their size, be allocated to meiofauna or macrofauna are also united by other ecological responses, nor that they constitute independent ecological units. As Magni et al. (2022) stress, it would be more appropriate to treat the two notionally different faunas as a single dataset and merging them would make a more comprehensive and robust tool for the examination of the ecology of estuarine systems.

## Author Contributions

**R. S. K. Barnes:** conceptualization (equal), data curation (equal), formal analysis (equal), investigation (equal), methodology (equal), project administration (equal).

## Funding

The Department of Science and Innovation/National Research Foundation Research Chair in Shallow Water Ecosystems (UID 84375) provided funding for field work and analysis; no grants from external funding agencies in the public, commercial, or not‐for‐profit sectors were received.

## Ethics Statement

This study was conducted in the Garden Route National Park in accordance with all regulations and requirements appertaining to research in a South African National Park, and within the guidelines of the Russell and Burch (1959) Replacement, Reduction, and Refinement Framework and those for the care and use of animals and habitats set out by Schlacher et al. (2025).

## Conflicts of Interest

The author declares no conflicts of interest.

## Data Availability

All original meiofaunal data used in this comparison are available at https://doi.org/10.5061/dryad.r2280gbtd; the availability of macrofaunal data already in the public domain is listed in the Appendix A.

## Appendix A

Representative number of macrofauna m^−2^ in the major component taxa at the ten disparate sites representing the Knysna estuarine bay (assembled from the data of Barnes, R.S.K., 2022, Biodiversity differentials between seagrass and adjacent bare sediment change along an estuarine gradient. Mendeley Data, VI, doi: 10.17632/nmwsrpd738.1; Barnes, R.S.K., Claassens, L., 2023, Subtidal vs. intertidal seagrass assemblages along the Knysna Estuary. Mendeley Data, VI, doi: 10.17632/nj2mvv8fn5.1; Barnes, R.S.K., 2024, Warm‐temperate flood‐tidal sand‐delta macrofauna. Mendeley Data, VI, doi: 10.17632/pwj6rsd2b3.1; and Barnes, R.S.K., 2026, Seagrass vs. bare sand conytrasts in absence of bioturbation. Mendeley Data. VI, doi: 10.17632/95jg8rckv7.1). Key: Biv, bivalve molluscs; Cyl, the ostracod ?*Cylindroleberis*; Deca, decapod crustaceans; Ech, echinoderms; Err, errant polychaetes; Gast, gastropod molluscs; Nem, polyclad flatworms and nemertines; Pera, peracaridan crustaceans; Sed, sedentary polychaetes.

**Table:**

Compartment	Habitat	Nem	Err	Sed	Cyl	Pera	Deca	Gast	Biv	Ech
Mouth	Seagrass	4	153	223	165	195	184	939	51	76
Mouth	Bare	40	201	2000	105	444	35	785	59	0
Bay	Seagrass	5	85	372	184	145	93	160	80	76
Bay	Bare	0	332	145	16	70	5	7	4	0
Lagoon	Seagrass	2	121	1116	118	95	133	241	198	107
Lagoon	Bare	1	91	1408	37	42	33	127	64	1
Estuary	Seagrass	1	134	251	0	38	9	145	206	1
Estuary	Bare	0	50	87	0	14	2	58	121	0
Backwaters	Seagrass	24	37	38	0	152	123	5405	5	52
Backwaters	Bare	26	484	809	0	763	66	8664	11	2
